# The Different Stages of Vestibular Neuritis from the Point of View of the Video Head Impulse Test

**DOI:** 10.4081/audiores.2020.248

**Published:** 2020-08-24

**Authors:** Leonardo Manzari, Domenico Graziano, Marco Tramontano

**Affiliations:** 1 MSA ENT Academy Center, Cassino, Italy; domenicograziano96@gmail.com; 2 IRCCS Santa Lucia Foundation, Rome, Italy; m.tramontano@hsantalucia.it; 3 Department of Movement, Human and Health Sciences, University of Rome “Foro Italico”, Interuniversity Centre of Bioengineering of the Human Neuromusculoskeletal System, P.zza Lauro de Bosis 15, 00135 Roma, Italy

**Keywords:** video Head Impulse Test, Vestibular Neuritis, Vestibulo-Ocular Reflex, Saccade, anticompensatory saccades

## Abstract

Vestibular neuritis (VN) is one of the most common causes of acute vestibular syndrome (AVS). Quantifying the vestibulo-ocular reflex (VOR) gain by the video Head Impulse Test (vHIT) could provide useful information to diagnose VN. This study aims to retrospectively evaluate the VOR gain values during the acute and subacute stages of the VN and to correlate these values with the patients’ dizziness-related handicap. Medical record of 28 patients with VN were reviewed. Patients were assigned to two groups according to the time since the acute vestibular syndrome (AVS). One group with patients assessed within seventy-two hours since the AVS (AVSg) and one group with patients evaluated from four days to six weeks since the AVS (PAVSg). VOR gain was evaluated in all selected patients and correlated to Dizziness Handicap Inventory (DHI). Significant differences were found in the between-subjects analysis in DHI score (*p* = 0.000) and in the ipsilesional hVOR gain values (*p* = 0.001). The correlation analysis showed significant results (*p* = 0.017) between DHI score (40 ± 16.08) and ipsilesional VOR gain (0.54 ± 0.09) in the PAVSg. Patients evaluated within 72 h since the AVS showed anticompensatory saccades (AcS) turning the head toward the contralesional side. Patients with unilateral Superior VN (SVN) could have dissimilar hVOR gain values and DHI score according to the damage of the VIII pair of cranial nerves. AcS in the contralesional side is a sign of acute phase in patients with unilateral SVN.

## 1. Introduction

Vestibular neuritis (VN) is a clinical entity that refers to an otoneurological disorder characterized by acute, prolonged vertigo of peripheral origin, one of the most common causes of acute vestibular syndrome (AVS) and affects predominantly Vestibulo Ocular Reflex (VOR) [1].

The acute phase, which usually coincides with the first seventy-two hours following the onset of symptoms [2], is defined by severe rotatory vertigo with nausea and vomiting where only vestibular function and not hearing is affected [1]. These patients fall into three main groups, suggesting that neuritis may affect the entire vestibular nerve or just one of the branches of it 3–6, and certainly the most frequent clinical entity is that which afflicts the upper compartment of the eighth pair of cranial nerves, called Superior Vestibular Neuritis (SVN) [3].

VN diagnosis, especially in AVS, is a great challenge for the clinician. Nystagmus characteristics, other oculomotor findings, and the head impulse test are commonly used in combination often complemented with imaging. Clinicians can identify the classical signs and symptoms (vertigo is the main one) which are dependent on the absent function of the affected vestibular labyrinth differentiating strokes from peripheral vestibular disorders [2]. 

After its clinical validation vs dual magnetic scleral search coil [4] video Head Impulse Test (vHIT) constitutes the diagnostic clinical gold standard of the horizontal and vertical VOR. Thus, during a brief, passive, unpredictable, horizontal head turns towards the healthy ear, the subject maintains fixation on an earth-fixed target while the patient with reduced unilateral horizontal or vertical canal function fails to maintain fixation on an earth-fixed target and so makes corrective (overt or covert) saccades during or at the end of the head rotation in order to regain fixation [5]. The saccades that occur during head movement are known as covert and those that occur following after the end of the head movement, as overt saccades [6].These saccades are a substitution sign of the reduced VOR dynamic function. This paradigm is also called Head Impulse Paradigm (HIMP). A new complementary test paradigm was recently proposed to be an indicator of vestibular function [7]. This paradigm has been named suppression head impulse test (SHIMP), where the subject is asked to look at a head-fixed target rather than at the earth-fixed target used in HIMP during the passive head turn. Healthy subjects make a corrective saccade (a “SHIMPs” saccade), whereas patients at the time of acute peripheral vestibular loss do not [6]. SHIMPs paradigm gives more precise information on the VOR gain compared to HIMPs because the evaluation of the gain is not affected by covert saccades [7].

Despite the evidence in diagnosing vestibular neuritis [8,9] through the vHIT, some relevant clinical aspects are poorly understood. Instead, it is still unclear which stage of the disease the VOR gain values correspond to, and above all, it is not clear if the VOR gain values are related to disability complained by patients. Our hypothesis is that the VOR gain values might be dissimilar according to the damage of the VIII pair of cranial nerves in the real acute stage (within seventy-two hours) or the subacute stage (seventy-two hours six weeks) and on patients’ perception of handicap due to the dizziness.

This study aims to retrospectively evaluate the VOR gain values during the acute and subacute stages of the VN and to correlate these values with the patients’ perception of handicap due to the dizziness.

## 2. Materials and Methods

### 2.1. Study Design

This is a retrospective study aimed to evaluate the VOR gain values during the acute and subacute stages of the VN and to correlate these values with the subjective symptoms measured by the Dizziness Handicap Inventory (DHI) score. All procedures contributing to this work comply with the ethical standards of the relevant national and institutional guidelines on human experimentation and with the Helsinki Declaration of 1975. The study was carried out according to the Strengthening the Reporting of Observational Studies in Epidemiology (STROBE) guidelines. All patients gave written consent to publish the results obtained from their clinical examinations and instrumental tests.

### 2.2. Setting

Medical records of patients with a diagnosis of VN in the early stages of the disease (from the first hours to six weeks since the AVS) and still symptomatic who were admitted to the ENT MSA Academy Center from 2014 to 2019 were reviewed. 

### 2.3. Participants

The inclusion criteria were diagnosis of VN in the early phase (within six weeks since the AVS) and patients who had not undergone pharmacological treatment for VN. We excluded the medical records of patients who showed other vestibular diagnosis (more than six weeks since the AVS, Meniere disease, bilateral vestibular loss, vestibular migraine, BPPV, etc.), somatic or psychiatric disorders, presence of neurological diseases. All patients were undergone to a vestibular assessment that included a self-assessment inventory with DHI, an assessment of horizontal and vertical semicircular canals with bedside Head Impulse Test + video-Head Impulse Test, Air Conducted Sound, and Bone Conducted Vibration Cervical and Ocular VEMPs.

VN was diagnosed on the following criteria: (a) a history of acute onset of severe, prolonged, rotatory vertigo, nausea, and postural imbalance; (b) on clinical examination the presence of horizontal spontaneous nystagmus with a rotational component toward the unaffected ear (fast phase) without evidence of a central vestibular lesion; (c) abnormal bed-side HIT showing an ipsilateral deficit of the horizontal semicircular canal [1]; (d) alterations in the cervical and ocular VEMPs results compatible with the diagnosis of VN or SVN and absence of neurological signs; (e) an MRI of the brain that showed no lesions that could account for any vestibular disturbance and performed at least forty-eight hours after the AVS onset [10]. Patients were assigned to two groups according to the onset of symptoms. One group for patients assessed within seventy-two hours since the AVS (AVSg) and the second one for patients assessed in the post-acute phase (PAVSg) from seventy-two hours to six weeks since the AVS. The AVSg hVOR values in the post-acute phase were also reported.

### 2.4. Dizziness Handicap Inventory

The patients’ perception of handicap due to the dizziness of all VN patients was assessed by the DHI. The DHI is a self-assessment inventory, including 25 questions to evaluate self-perceived activity limitation and restriction resulting from dizziness [11].

### 2.5. Video Head Impulse Test

The function of the horizontal semicircular canals was measured using horizontal vHIT (OtosuiteV^®^, GN Otometrics, Denmark) as previously described during HIMP. Subjects were instructed to fixate an earth-fixed dot on the wall at 1m distance in front of them. Room lighting conditions were adjusted to ensure that the pupil was small and the pupil image was not affected by reflections in the pupil image at any point in the range of the head movement. At each testing epoch the clinician (L.M.) applied about 20 brief, rapid, horizontal head turns (head impulses) to each side, always starting from centre, with unpredictable timing and direction with minimal bounce-back or overshoot at the end of the head impulse: each head impulse was “turn and stop”. The amplitude of the head rotation was about 10–15 deg, and the peak head velocity of the impulse was about 140–220 deg/s, with angular accelerations of between about 3000 deg/s^2^ and 5000 deg/s^2^. Eye velocity and head velocity were recorded for each head turn. The usual measure of the adequacy of the vestibulo-ocular reflex, VOR slow phase in the HIMP paradigm is gain. Gain value <0.76 identifies the affected side of Unilateral VN with 100% sensitivity (48–100) and 100% specificity (74–100). VOR gain represents the ratio of the area under the curve (AUC), during HIMP of 1.0 (0.81–1.0, *p* < 0.0001) [10]. SHIMPs testing procedure was exactly the same as for HIMPs with one exception. Participants were instructed to fixate a head-fixed target—a laser spot projected on the wall at 90 cm distance in front of them projected by a head-mounted laser [6]. This spot moved with the head, and during testing it appeared to subjects that they were looking at a dot which unexpectedly jumped around. At least ten impulses were delivered to left and right sides, respectively. To avoid anticipation, the head turn always started from the center. Eye velocity, head velocity and percentage of impulses containing saccades were recorded and evaluated in each head rotation. VOR gain was calculated during SHIMPs paradigm because the evaluation of the gain is not affected by covert saccades. SHIMP gains (<0.66) identified the affected side of Unilateral VN with 100% sensitivity (48–100) and 100% specificity (74–100) and an AUC of 1.0 (0.81–1.0, *p* < 0.0001) [6,7]. Compensatory saccades in the affected side were defined as saccades in the direction of the eye movement while Anticompensatory Saccades (AcS) or quick eye movement in the contralesional side were defined as saccades (peak velocity above 50°/s) in the direction of the head movement. AcS start was at 10°/s, latency was the difference between head impulse and AcS start. AcS occurrence rate was the percentage of impulses with AcS [12].

Demographic and clinic characteristics are reported in Table 1.

## 3. Statistical Analysis

All the statistical analyses were carried out using the IBM SPSS Statistic Software Version 23, IBM Corp., Armonk, NY, U.S.A. Data were reported in terms of means and standard deviations. The Mann-Whitney U-test was used to compare DHI data between groups. Independent-samples T-test was used for VOR Gain data in order to compare values between the two groups. The Pearson’s Correlation Coefficient was calculated between DHI and Ipsilesional VOR gain data.

## 4. Results

Twenty-eight patients with a diagnosis of unilateral superior VN (SVN) met the inclusion criteria and were enrolled in the study. Altered asymmetry ratio values compatible with the diagnosis of VN of ocular Air Conducted Sound and Bone Conducted Vibration VEMPs were found in all participants, whereas the asymmetry ratio values of cervical VEMPS were in the normal ranges. 

VOR gain in the AVSg was 0.39 ± 0.17 and 0.54 ± 0.09 in the PAVSg, the DHI score was 78.80 ± 7.28 in the AVSg and 40 ± 16.08 in the PAVSg. Significant differences were found in the between-subjects analysis in DHI scores (*p* = 0.000) and in the ipsilesional hVOR gain values (*p* = 0.001), no differences were found in the contralesional VOR gain values (*p* = 0.601). Nine out of fifteen patients of AVSg evaluated in the post-acute phase showed a statistical significant difference from the baseline (*p* < 0.05) in the hVOR values (0.57 ± 0.25).

**Figure 1:**
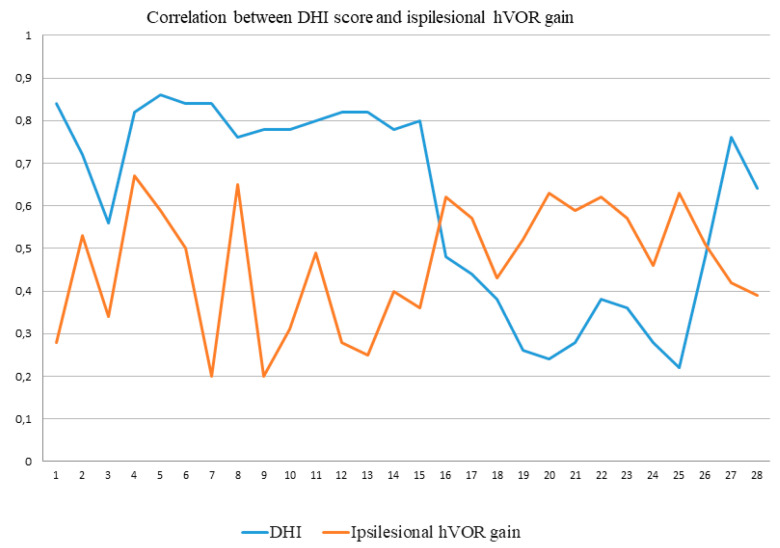
Graphical representation of the correlation between DHI scores and ipsilesional hVOR gain in the two groups. Data from 1 to 15 referred to the AVSg, data from 16 to 28 referred to the PAVSg. DHI = Dizziness Handicap Inventory; hVOR = horizontal vestibulo-ocular reflex; patient IDs are indicated on the x-axis; AVSg = Acute vestibular syndrome group; PAVSg = Post-acute vestibular syndrome group.

The correlation analysis showed significant results (*p* = 0.03) between DHI score (40 ± 16.08) and ipsilesional VOR gain (0.54 ± 0.09) in the subacute PAVSg as reported in Figure 1.

All AVSg patients and three of the PAVSg presented AcS on the contralesional side. All patients of the AVSg evaluated in the post-acute phase did not show more AcS.

## 5. Discussion

This retrospective study aimed to evaluate the VOR gain values during the acute and subacute stages of the VN. Great emphasis in the last few years was given about the changes in the timing and pattern of corrective saccades during the different stages of VN [6,7,8,13].

On the contrary, we focused our attention on other aspects of the clinical course observation timing as well as on the valid indicator of semicircular canal function (eye movements) in response to small, brief, fast, unpredictable horizontal head turns (head impulses) angular acceleration up to 4000°/s^2^. 

Firstly our findings show a significant difference of hVOR gain values in the two groups (Table 1), suggesting that the hVOR gain values can change, just like saccades pattern, during the different stages of VN. These clinical data are important because it represents an important indicator of lesion evolution in the SCC dynamic function, especially at the time of the attack. Our findings can provide useful clues about the clinical timing and progress of recovery from the lesion through the value of the hVOR gain which can, therefore, accompany changes and now known evolution of the saccades pattern that allows the patient to stabilize vision on the retina.

Testing patients within seventy-two hours and after this period allows highlighting an increase in the hVOR gain as previously described but in a longer observation period [8,9,14]. Furthermore, correlating the hVOR gain with the DHI score in the different phases emerged that the subjective symptoms are significantly lower in the PAVSg as reported in Figure 1. The data could therefore also correlate with the modifications in VOR gain values (increase) which in the early stages is strongly and suddenly compromised if compared with the contralesional side. Of course an increase of VOR gain can predict an improvement of the clinical condition, but possibly other factors may play a role, as the evolution of the saccades pattern, i.e., from overt to covert saccades and as recently published [13], the reappearance of SHIMPs saccades.

The finding of an increase in the VOR gain could be the prelude to a restitutio ad integrum [9].

**Figure 2:**
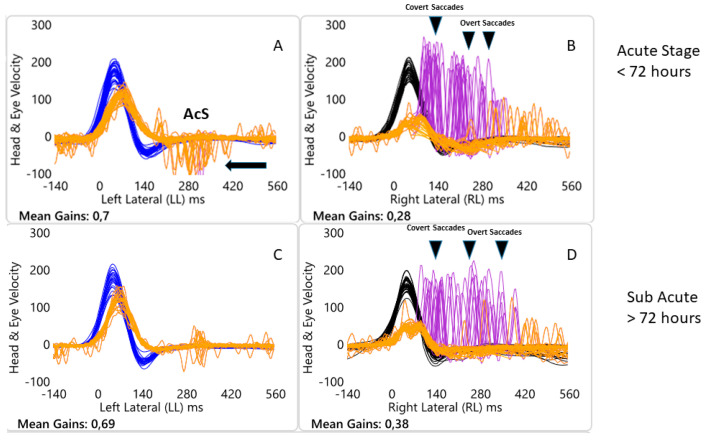
Objective measures of horizontal semicircular canal function at 2 testing occasions for one patient from AVS group with acute unilateral (right) vestibular neuritis: occasion 1 (**A**,**B**) and at succeeding time thereafter (**C**,**D**). Each panel shows a superimposed time series of head velocity (blue for the left impulses and black for the right impulses) and the corresponding eye velocity (orange) for the tests of horizontal canal dynamic function using vHIT. The signs of head velocity for leftward impulses and of eye velocity for rightward impulses have been inverted to allow for easier comparison. Normal horizontal VOR gains are about 0.7–1.0 [4]. For rotations to the affected side, eye velocity is substantially less than the corresponding head velocity during the head turn so the VOR is significantly less, VOR gain is 0.28, then for head turns to the healthy side, VOR gain is 0.7. There is a shower of compensatory (Covert + Overt) saccades during and at the end of the head turn (black arrow). While when the head is turned to the left contralesional side, anti compensatory saccades (AcS) eye movements in the direction of the head movement can be observed (grey arrow) at the time of the attack. On succeeding testing occasion, two months later, for head turns to the affected side the slow phase eye velocity improves and the corrective saccades are still the same, VOR gain is 0.38, for head turns to the healthy side the slow phase velocity is quite the same while anti-compensatory saccades disappear.

Secondly, VOR destabilization process that occurs during SVN can also be associated with the presence of AcS (Figure 2) [12], that can be highlighted in particular, by turning the head towards the healthy side, in the early stages of the disease and which tend to be absent in the PAVSg (73%) as well as disappear in all patients of VASg at second evaluation (Figure 2). 

About this phenomenon, AcS presence could be associated, in our opinion, as a sign of peripheral deficit due to the contribution to the process of the quick eye movements linked to the failure of the affected side to contribute to the desired realization of the reflex. These saccadic movements are in the same direction of the head movement and it has been previously hypothesized that they are an indicator of peripheral injury [12]. Last but not least, considering our sample, we can hypothesize that this phenomenon could be an indicator of acute peripheral injury in the very early stages of the inflammatory process.

For this reason, why are AcS, in “early” vHIT evaluation, so potentially valuable especially as an indicator of VN acute stage? Following unilateral vestibular loss, it is known that the spontaneous nystagmus pattern is characterized by a slow phase of its horizontal component in the opposite direction of head rotation while the quick phase, rapid eye movement in the direction of head rotation, is directed away from the lesioned side, in the same direction of head rotation. 

It is also known: (a) quick phases and saccades are both rapid eye movements with similar kinematics characteristics and (b) there are two distinct neural circuits responsible for the genesis of horizontal eye movements. The first for the slow phase in response to head turns, generated by the direct three neurons pathway: receptor/vestibular nucleus/abducens motoneurons/extraocular eye muscles.

The second is instead responsible for the generation of a separate neural circuit involving a network of burst neurons and pause neurons in the brainstem extremely close to abducens nucleus [15]. This last circuit can be triggered by vestibular input thereby generating the quick phase of vestibular nystagmus during prolonged horizontal angular acceleration or by descending axons from the superior colliculus causing voluntary saccades but there is also evidence that input from neck afferents can also trigger this quick phase neural circuit [15].

In our patients, the horizontal slow phase mechanism was disabled at the acute stage (as shown by the very low gain VOR <72 h after the AVS onset, see Figure 2A,B) most probably because of the acute damage to the superior vestibular nerve component. However, the quick phase mechanism was unaffected as shown by the saccades at each head turn toward affected side and just like in the case of spontaneous nystagmus, when the head is passively and abruptly turned towards the healthy side, a quick phase (AcS), similar to that of spontaneous nystagmus directed away from the lesioned side, is generated. Essentially these quick eye movements seem to be related to the presence of spontaneous nystagmus. The latter in the acute phases of unilateral SVN can be reduced but not completely suppressed in the light. In fact, vHIT test is usually conducted not in darkness but in an illuminated environment. 

We acknowledge some limitations of the present study. First, we reported only the hVOR values of the patients with unilateral Superior VN but further studies could be carried out also in patients with complete VN. Another limitation is that patients of PAVSg were evaluated in a larger range of time with respect to AVSg but to the best of our knowledge there is not a scientific consensus on the time of VN phases (e.g., acute, post acute or chronic). Furthermore, a follow up is needed in further studies to better evaluate the true clinical evolution of AVS in these patients.

## 6. Conclusions

Our results demonstrate that hVOR gain and DHI score could be dissimilar according to the damage of the vestibular nerve in our VN sample and in relation to the time elapsed since the origin of the symptomatology reported by the patient. On the part of the clinician, this is of fundamental importance since the earliness of the diagnosis of peripheral nosological entities allows to plan an adequate diagnostic path. VN patients evaluated within seventy-two hours since the AVS show AcS in the contralesional side. The presence of these quick eye movements means a clinical sign of a very acute phase.

## Figures and Tables

**Table 1:** Demographic and clinical characteristics.

	Age (Years) ±SD	Sex n (%) F-M	DHI	Ipsilesional hVOR Gain	Contralesional hVOR Gain	AcS Contralesional n (%)
AVSg (n = 15)	55 ± 16.5	8 (54)–7(46)	78.80 ± 7.28	0.39 ± 0.17	0.91 ± 0.11	15 (100)
PAVSg (n = 13)	53.9 ± 18.2	6 (46)–7(54)	40 ± 16.08	0.54 ± 0.09	0.94 ± 0.25	10 (73)
*p* value	*p* = 0.436		*p* = 0.000 *	*p* = 0.001 *	*p* = 0.601	

AVSg: Acute Vestibular Syndrome group; PAVSg: Post Acute Vestibular Syndrome group; Mean±standard deviation; DHI: Dizziness Handicap Inventory; hVOR: horizontal canal Vestibulo Ocular Reflex; AcS: Anticompensatory saccades; * significant for *p* < 0.05.
